# Supplementary material for: Causal Relationship between Aging and Anorexia Nervosa: A White-Matter-Microstructure-Mediated Mendelian Randomization Analysis
Source: Biomedicines. 2024 Aug 16;12(8):1874. doi: 10.3390/biomedicines12081874 (PMC11351342; doi:10.3390/biomedicines12081874)
Supplement: Supplementary file 1 [file biomedicines-12-01874-s001.zip › Supplementary Figure/Figure S4/Singe SNP plot of MR analysis for TL to ubm-b-1545.pdf]

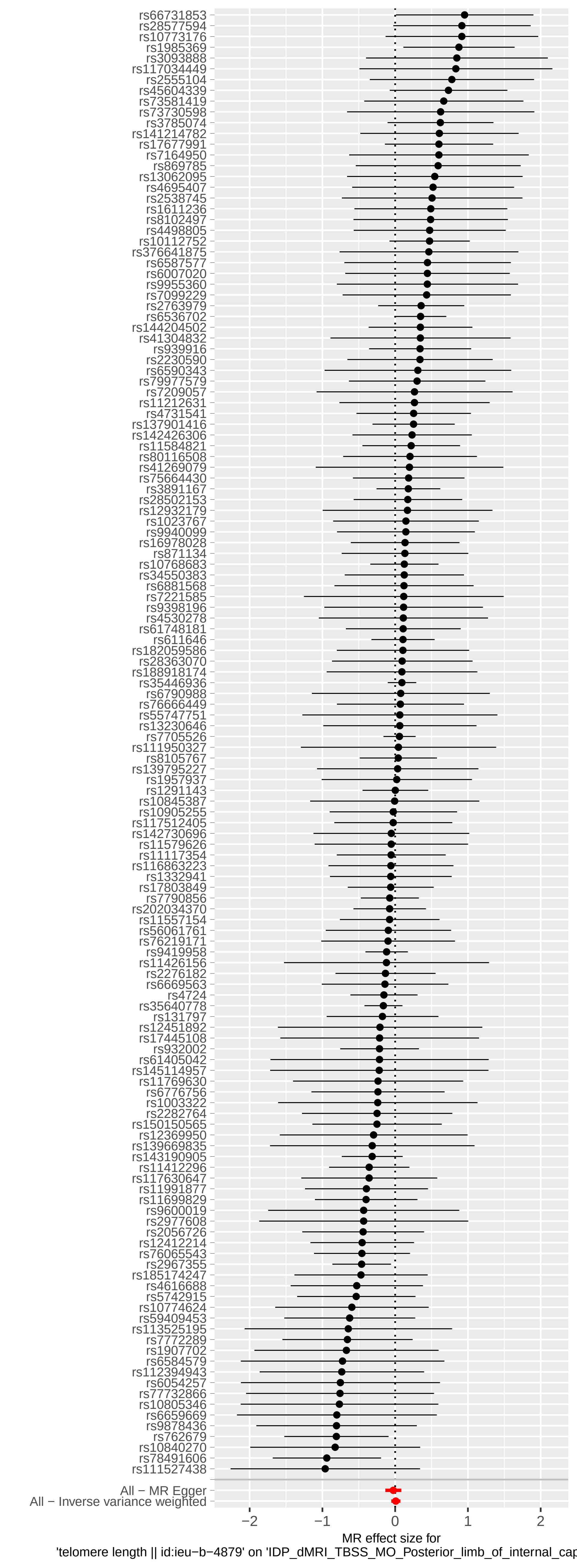

rs66731853
rs28577594
rs10773176
rs1985369
rs3093888
rs117034449
rs2555104
rs45604339
rs73581419
rs73730598
rs3785074
rs141214782
rs17677991
rs7164950
rs869785
rs13062095
rs4695407
rs2538745
rs1611236
rs8102497
rs4498805
rs10112752
rs376641875
rs6587577
rs6007020
rs9955360
rs7099229
rs2763979
rs6536702
rs144204502
rs41304832
rs939916
rs2230590
rs6590343
rs79977579
rs7209057
rs11212631
rs4731541
rs137901416
rs142426306
rs11584821
rs80116508
rs41269079
rs75664430
rs3891167
rs28502153
rs12932179
rs1023767
rs9940099
rs16978028
rs871134
rs10768683
rs34550383
rs6881568
rs7221585
rs9398196
rs4530278
rs61748181
rs611646
rs182059586
rs28363070
rs188918174
rs35446936
rs6790988
rs76666449
rs55747751
rs13230646
rs7705526
rs111950327
rs8105767
rs139795227
rs1957937
rs1291143
rs10845387
rs10905255
rs117512405
rs142730696
rs11579626
rs11117354
rs116863223
rs1332941
rs17803849
rs7790856
rs202034370
rs11557154
rs56061761
rs76219171
rs9419958
rs11426156
rs2276182
rs6669563
rs4724
rs35640778
rs131797
rs12451892
rs17445108
rs932002
rs61405042
rs145114957
rs11769630
rs6776756
rs1003322
rs2282764
rs150150565
rs12369950
rs139669835
rs143190905
rs11412296
rs117630647
rs11991877
rs11699829
rs9600019
rs2977608
rs2056726
rs12412214
rs76065543
rs2967355
rs185174247
rs4616688
rs5742915
rs10774624
rs59409453
rs113525195
rs7772289
rs1907702
rs6584579
rs112394943
rs6054257
rs77732866
rs10805346
rs6659669
rs9878436
rs762679
rs10840270
rs78491606
rs111527438
All − MR Egger
All − Inverse variance weighted
−2
−1
0
1
2
MR effect size for
'telomere length || id:ieu−b−4879' on 'IDP_dMRI_TBSS_MO_Posterior_limb_of_internal_cap
